# Cd‐Rich Alloyed CsPb_1‐_ *_x_*Cd*_x_*Br_3_ Perovskite Nanorods with Tunable Blue Emission and Fermi Levels Fabricated through Crystal Phase Engineering

**DOI:** 10.1002/advs.202000930

**Published:** 2020-06-17

**Authors:** Jie Guo, Yuhao Fu, Min Lu, Xiaoyu Zhang, Stephen V. Kershaw, Jia Zhang, Shulin Luo, Yanxiu Li, William W. Yu, Andrey L. Rogach, Lijun Zhang, Xue Bai

**Affiliations:** ^1^ State Key Laboratory of Integrated Optoelectronics and College of Electronic Science and Engineering Jilin University Changchun 130012 China; ^2^ State Key Laboratory of Superhard Materials and College of Physics Jilin University Changchun 130012 China; ^3^ State Key Laboratory of Integrated Optoelectronics Key Laboratory of Automobile Materials of MOE and College of Materials Science and Engineering Jilin University Changchun 130012 China; ^4^ Department of Materials Science and Engineering Centre for Functional Photonics (CFP) City University of Hong Kong Hong Kong SAR China; ^5^ Department of Chemistry and Physics Louisiana State University Shreveport LA 71115 USA

**Keywords:** alloyed perovskites, blue emission, crystal phase engineering, Fermi levels, nanorods

## Abstract

One‐dimensional semiconductor nanostructures have already been used for a variety of optoelectronic applications. Metal halide perovskites have emerged in recent years as promising high‐performance optoelectronic materials, but reports on 1D nanorods (NRs) of all‐inorganic halide perovskites are still scarce. This work demonstrates a synthetic strategy toward cesium‐based inorganic perovskite NRs by exploiting composition‐controlled crystal phase engineering. It is accomplished for Cd‐rich mixed‐cation CsPb_1‐_ *_x_*Cd*_x_*Br_3_ nanocrystals, where the initial 1D hexagonal perovskite phase drives the growth of the 1D NRs, as supported by first‐principles calculations. The band gaps of the resulting NRs are tunable by varying the Cd‐content, and the highly uniform CsPb_0.08_Cd_0.92_Br_3_ NRs (with an average length of 84 nm and width of 16 nm) exhibit a true blue‐color emission centered at 460 nm, with a high quantum yield of 48%. Moreover, this work also demonstrates the tunability of the Fermi levels in the films made of CsPb_1‐_ *_x_*Cd*_x_*Br_3_ alloyed nanocrystals, where samples with highest Cd content show an increase of the electron concentration and a related increase in the conductivity.

## Graphical abstract

It is shown that upon incorporation of sufficient levels of Cd^2+^ cations, the isotropic 3D cubic lattice of CsPbBr_3_ nanocrystals gives way to a transformation to the 1D anisotropic hexagonal lattice, which allows 1D growth of alloyed CsPb_1‐_ *_x_*Cd*_x_*Br_3_ nanorods to be realized whose bandgaps can be tailored all across the blue spectral range from 2.40 eV for x = 0.10 to 2.74 eV for x = 0.96.

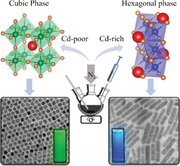

Nanoscale semiconductors with a one‐dimensional (1D) morphology such as nanowires (NWs) and nanorods (NRs) have been extensively studied for their optical/electrical properties, and a number of applications in lasing, photonics and next‐generation backlighting for liquid crystal displays have been explored.^[^ ^1^ ^]^ Recent studies revealed all‐inorganic (cesium‐based) lead halide (CsPbX_3_; X = Cl, Br, I) perovskite nanocrystals (NCs) to be excellent photonic and optoelectronic materials owing to their long carrier lifetimes, large absorption cross sections, near‐unity photoluminescence quantum yields (PLQYs), and PL color tunability over the whole visible spectrum.^[^ ^2^ ^]^ In the past few years, impressive progress has been made in the synthesis of high‐quality CsPbX_3_ perovskite NWs with well‐defined morphology and tunable optical properties, which was accomplished by vapor‐phase epitaxial growth,^[^ ^3^ ^]^ solution‐phase synthesis,^[^ ^4^ ^]^ template‐assisted synthesis,^[^ ^5^ ^]^ nanoparticle assembly,^[^ ^6^ ^]^ and hot‐injection methods.^[^ ^7^ ^]^ However, the advancements of CsPbX_3_ perovskite NRs have been rather slow as compared with their NW counterparts, with only a few examples of successful synthetic procedures published recently.^[^ ^8^ ^]^ On the other hand, many conventional semiconductor NCs such as cadmium selenide are intrinsically capable of being anisotropic materials, which can easily grow into a rod‐shaped morphology under appropriate synthesis conditions, owing to their wurtzite crystal structure with an extended *c* axis.^[^ ^9^ ^]^ Therefore, the rod‐shaped morphology should in principle be achievable in CsPbX_3_ perovskite materials by intentionally designing a 1D anisotropic crystallographic structure. However, CsPbX_3_ NCs primarily adopt the cubic or orthorhombic crystal phase, whose lattice structure exhibits 3D interconnection of corner‐shared [PbX_6_]^4−^ octahedra with the Cs^+^ cations filling the voids created by four neighboring [PbX_6_]^4−^ octahedra (Figure S1, Supporting Information).^[^ ^10^ ^]^ At the same time, some other kind of perovskite crystals such as CsCdX_3_ possess the 1D crystallographic structure, where [CdX_6_]^4–^ octahedra sharing opposite faces form infinite linear chains along the crystallographic *c* axis (Figure S1, Supporting Information).^[^ ^11^ ^]^ Those materials have not attracted much attention in the optical research community due to their low PLQYs.^[^ ^12^ ^]^ At the same time, many studies have pointed out that alloying of semiconductor materials offers a powerful approach to tune their crystalline phase as well as to tailor their optical and electronic properties.^[^ ^2^, ^13^ ^]^ We, thus, anticipated that a 1D crystallographic structure, which has been previously reported for CsCdX_3_ perovskites, could be realized in CsPb_1‐x_Cd_x_Br_3_ alloyed NCs, triggering the formation of a rod‐shaped morphology.

To validate this assumption, we synthesized a series of alloyed CsPb_1‐_ *_x_*Cd*_x_*Br_3_ NCs with different Pb/Cd ratios. We demonstrate how the crystallographic structure of these alloyed NCs can be converted from 3D cubic to 1D hexagonal structure by deliberately varying the CdBr_2_/PbBr_2_ molar ratio in the synthesis (**Figure** **1**). The 1D hexagonal crystal structure similar to that of the CsCdBr_3_ NRs could be obtained at higher CdBr_2_ amount, while at lower CdBr_2_ amount, the resulting CsPb_1‐_ *_x_*Cd*_x_*Br_3_ alloyed NCs still maintained the 3D cubic crystallographic structure of CsPbBr_3_ (Figure 1). With the realization of the 1D hexagonal lattice, the rod‐like shape has been successfully achieved for the alloyed Cd‐rich CsPb_1‐_ *_x_*Cd*_x_*Br_3_ NCs (Figure 1). We also demonstrate how the band gaps and the Fermi levels change in the alloyed CsPb_1‐_ *_x_*Cd*_x_*Br_3_ NRs compared with their CsPbBr_3_ counterparts, which results in a blue‐shift of the emission band as well a stronger n‐type conductivity. In particular, CsPb_0.08_Cd_0.92_Br_3_ NRs exhibit blue‐color emission centered at 460 nm with PLQYs of 48%, and their conductivity is larger than either in CsPbBr_3_ NCs or CsCdBr_3_ NRs owing to the considerable increasing of the electron concentration.

**Figure 1:**
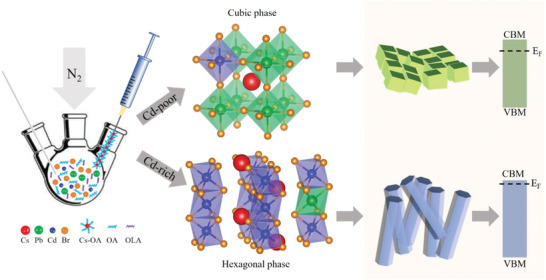
Illustration of the synthetic strategy allowing us to modulate the crystallographic structure of alloyed CsPb_1‐_ *_x_*Cd*_x_*Br_3_ perovskite NCs by controlling the CdBr_2_/(CdBr_2_+PbBr_2_) molar ratios in a hot‐injection synthetic process. In the Cd‐poor synthetic environment, the alloyed NCs maintain the 3D cubic crystallographic structure of CsPbBr_3_, while under Cd‐rich conditions, they adopt the 1D hexagonal crystallographic structure. This results in a different morphology (nanocubes vs NRs) and changes of the band gaps and the Fermi levels of the resulting NCs.

We initially evaluated the possibility of the formation of a 1D crystallographic structure in the CsPb_1‐_ *_x_*Cd*_x_*Br_3_ perovskite by performing first‐principles calculations based on the density functional theory DFT, by considering the enthalpies of the materials with a different Cd content (*x*). Details on the calculations of 3D cubic and 1D hexagonal crystallographic structures are given in the theoretical calculation section in the Supporting Information. As shown in **Figure** **2**a, at the lower Cd contents (*x* ≤ 0.20), the enthalpy per atom is smaller for the 3D cubic crystal structure than for the 1D hexagonal one, indicating that CsPb_1‐_ *_x_*Cd*_x_*Br_3_ perovskite would adopt the 3D cubic structure to maintain the structural stability. For the higher Cd contents (≥0.25), the enthalpy per atom for the 1D hexagonal crystallographic structure decreases gradually, implying the possibility of conversion from 3D cubic to 1D hexagonal structure. For the Cd content higher than 0.85, the enthalpy per atom for 1D hexagonal structure becomes 20 meV smaller than that for the 3D cubic structure, which means that the CsPb_1‐_ *_x_*Cd*_x_*Br_3_ perovskite would certainly tend to form 1D hexagonal crystallographic structure at such a Cd‐rich level.

**Figure 2:**
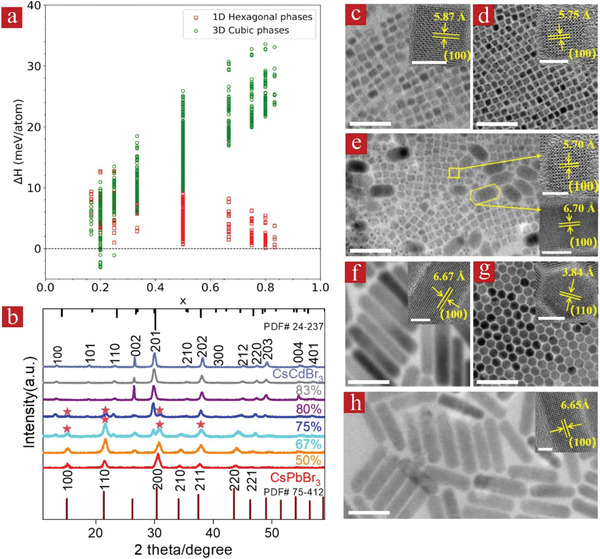
a) Calculated formation enthalpies, ΔH (in meV per atom), of the alloyed CsPb_1‐_ *_x_*Cd*_x_*Br_3_ (0 < *x* < 1) NCs as a function of the Cd content (*x*). b) XRD patterns of the CsPbBr_3_ NCs (*x* = 0) and CsCdBr_3_ NRs (*x* = 1), and alloyed CsPb_1‐_ *_x_*Cd*_x_*Br_3_ NCs with different Cd contents (*x*). The line spectra at the top and the bottom provide reference bars for the hexagonal phase (PDF #24‐237) and cubic phase (PDF #75‐412), respectively. c) TEM image of CsPbBr_3_ NCs. d–h) TEM images of alloyed CsPb_1‐_ *_x_*Cd*_x_*Br_3_ NCs with *x* values of d) 0.10, e) 0.56, f,g) 0.92, and h) 0.96. Corresponding HRTEM images are shown in the insets of frames of (c–h). Scale bars are 50 nm for the main frames and 5 nm for the insets, respectively.

Following these predictions from the theoretical calculations, we synthesized a series of CsPb_1‐_ *_x_*Cd*_x_*Br_3_ (0 < *x* < 1) NCs by varying the CdBr_2_/(CdBr_2_+PbBr_2_) molar ratio in the synthesis processes (details are shown in the Experimental Section, Supporting Information). The actual Cd content (*x*) on the B‐site of the alloyed CsPb_1‐_ *_x_*Cd*_x_*Br_3_ perovskite NCs has been identified by inductively coupled plasma mass spectrometry (ICP‐MS), and the values of *x* were determined as 0.10, 0.20, 0.56, 0.92, and 0.96 for the CdBr_2_/(CdBr_2_+PbBr_2_ ) precursor molar ratios of 50%, 67%, 75%, 80%, and 83%, respectively. For comparison, bare CsPbBr_3_ cubic‐shaped NCs and CsCdBr_3_ NRs were also synthesized in a similar way via the hot injection procedure^[^ ^14^ ^]^ (see Supporting Information for details). The crystal structures of alloyed CsPb_1‐_ *_x_*Cd*_x_*Br_3_ NCs, CsPbBr_3_ NCs, and CsCdBr_3_ NRs were characterized by X‐ray diffraction (XRD). XRD patterns can be indexed to the cubic perovskite structure (JCPDS No. PDF# 75‐412) for the CsPbBr_3_ NCs, and to the hexagonal perovskite structure (JCPDS No. PDF# 24‐237) for the CsCdBr_3_ NRs, as expected. We note that XRD reflexes of the CsCdBr_3_ sample exhibit some slight deviations compared with the reference bars, which may be ascribed to the influence of surface ligands on those NCs, affecting the lattice parameters of the core. When 50% molar ratio of the CdBr_2_ precursor was used in the synthesis, the resulting CsPb_0.90_Cd_0.10_Br_3_ NCs still retained the same cubic phase as the bare CsPbBr_3_ NCs, while all the diffraction peaks slightly shifted towards higher diffraction angles due to the substitution of larger (1.19 Å) Pb^2+^ cations by a proportion of Cd^2+^ cations with significantly smaller ionic radius (0.93 Å) (yellow curve in Figure 2b; Figure S2, Supporting Information).^[^ ^15^ ^]^ With an increasing amount of CdBr_2_ precursor, the evolution from the cubic to the hexagonal phase was observed in the alloyed NCs with CdBr_2_ precursor molar ratios of 67% (CsPb_0.80_Cd_0.20_Br_3_ NCs) and 75% (CsPb_0.44_Cd_0.56_Br_3_ NCs) (cyan and blue curves in Figure 2b). In these two samples, although several peaks (noted by a star in Figure 2b) related to the cubic structure still existed, a series of new diffraction peaks associated with the hexagonal phase of CsCdBr_3_ NRs (PDF#24‐237) emerged. The complete conversion from the cubic to the hexagonal phase took place when the CdBr_2_ molar ratio reached 80%; for the resulting CsPb_0.08_Cd_0.92_Br_3_ NCs, the positions of diffraction peaks were identical with those of the hexagonal CsCdBr_3_ NRs. There were no other additional diffraction peaks besides those of the hexagonal phase when we further increased the amount of CdBr_2_ precursor in the reaction. These results indicate that the large excess of Cd^2+^ cations in the reaction environment is indeed the requirement to achieve the 1D hexagonal crystallographic structure of the alloyed CsPb_1‐_ *_x_*Cd*_x_*Br_3_ perovskite NCs, which is well‐consistent with the theoretical predictions. To further explore the phase stability of both cubic and hexagonal perovskite structures, we have followed the change of XRD patterns of these two kinds of perovskite films in the air for 2 weeks, as shown in Figure S3, Supporting Information. Both 3D CsPbBr_3_ film (Figure S3a, Supporting Information) and 1D CsPb_0.08_Cd_0.92_Br_3_ film (Figure S3b, Supporting Information) maintained their original cubic phase crystal structure and hexagonal crystal structure, respectively, after 2 weeks, which illustrate that both kinds of perovskite films have reasonable phase stability. Careful examination of the XRD patterns in Figure S3b, Supporting Information, reveals that the intensity of the (002) peak became weaker upon storage over time for the 1D CsPb_0.08_Cd_0.92_Br_3_ film, implying a somewhat reduced degree of crystallinity. At the same time, the intensity of diffraction peaks of the 3D CsPbBr_3_ film remained unchanged, which points out that the 3D cubic crystal structure has a better stability.

The Pb/Cd alloyed structure of the perovskite NCs was further identified through analysis of X‐ray photoelectron spectra (XPS) (Figures S4 and S5, Supporting Information). We have taken two typical samples, that is, the CsPb_0.90_Cd_0.10_Br_3_ NCs with the 3D cubic structure and CsPb_0.08_Cd_0.92_Br_3_ NRs with the 1D hexagonal structure, and compared them with non‐alloyed CsPbBr_3_ NCs and CsCdBr_3_ NRs, respectively. Binding energies associated with Cs 3d, Pb 4f, and Br 3d in CsPb_0.90_Cd_0.10_Br_3_ NCs increased as compared with CsPbBr_3_ NCs, which confirms that the Cd^2+^ cations have replaced Pb^2+^ cations on the B‐site of the CsPbBr_3_ host, forming the alloyed structure (Figure S4, Supporting Information).^[^ ^10b^ ^]^ At the same time, the XPS peaks shifted towards lower binding energies in the CsPb_0.08_Cd_0.92_Br_3_ NCs as compared with CsCdBr_3_, thus further confirming formation of an alloyed structure (Figure S5, Supporting Information).^[^ ^2^, ^16^ ^]^

We have further investigated the morphology of the alloyed CsPb_1‐_ *_x_*Cd*_x_*Br_3_ NCs by transmission electron microscopy (TEM) (Figure 2c–h). For the CdBr_2_ molar ratio of 50%, the resulting CsPb_0.90_Cd_0.10_Br_3_ NCs were rather monodisperse, and exhibited a cubic shape similar to the CsPbBr_3_ NCs (Figure 2c,d; Figure S6a,b, Supporting Information). High‐resolution TEM (HRTEM) images of the CsPb_0.90_Cd_0.10_Br_3_ and CsPbBr_3_ NCs displayed lattice fringes of the (100) planes corresponding to the cubic crystal phase (insets of Figure 2c,d). With a further increase of the Cd content, TEM images of the CsPb_1‐_ *_x_*Cd*_x_*Br_3_ NCs showed the presence of two distinct shapes, namely small nanocubes and larger NRs (Figure 2e; Figure S7, Supporting Information), well consistent with the existence of the two crystal phases identified from the XRD patterns (Figure 2b). Representative HRTEM images of these samples revealed that the lattice distances of the nanocubes and NRs are 5.70 and 6.70 Å, respectively, which are well‐consistent with estimated distances of the (100) lattice planes in the cubic phase and (100) lattice planes in the hexagonal phase of the CsPb_0.44_Cd_0.56_Br_3_ alloyed NCs (Table S1, Supporting Information). As already mentioned from the XRD data, it appears that the conversion from the cubic to hexagonal phase becomes complete for the alloyed NCs synthesized with the use of a CdBr_2_ precursor molar ratio of 80% (Figure 2b), and indeed the TEM of this CsPb_0.08_Cd_0.92_Br_3_ sample showed the presence of highly uniform NRs with an average length of 84 nm and a width of 16 nm (Figure S6c,d, Supporting Information), corresponding to an aspect ratio of 5.25 (Figure 2f,g). HRTEM images of this sample demonstrate the existence of (100) lattice fringes extending along the rods (inset in Figure 2f), and (110) lattice fringes at the tips of the NRs (inset in Figure 2g), which are both associated with the hexagonal perovskite phase (Table S1, Supporting Information). With the further increase of the CdBr_2_ precursor amount, the NRs with a lower aspect ratio of 4.40 (average length of 57 nm and width of 13 nm) were obtained for the CsPb_0.06_Cd_0.94_Br_3_ NCs (Figure 2h). Large‐area TEM images of all samples have been collected and are shown in Figure S8, Supporting Information. With an increase of the Cd content, both rod‐like and hexagonal shapes gradually appeared on TEM images. When the Cd contents reached 0.92, the cubic shape completely disappeared (Figure S8e, Supporting Information). We note that for the CsPb_0.08_Cd_0.92_Br_3_, CsPb_0.04_Cd_0.96_Br_3_, and CsCdBr_3_ NRs (Figure S8e–g, Supporting Information), the width of the hexagons is the same as the width of the rods, which points out that the hexagonal contour represents a vertical view on the NRs, in these images.

Energy‐dispersive X‐ray (EDX) spectroscopy analysis and elemental mapping have been done on two representative samples, that is, the CsPb_0.08_Cd_0.92_Br_3_ NRs (shown in Figure S9a, Supporting Information), and CsPb_0.44_Cd_0.56_Br_3_ NCs that constitute a mixture of nanocubes and NRs (shown in Figure S9b, Supporting Information). The EDX analysis of these two samples shows that the amount of Cd^2+^ cations in the NR sample is 1.5 times higher than that in the shape‐mixed sample (Figure S10, Supporting Information), which corresponds very well with their structural formulae determined by ICP‐MS. Mapping images of the NR‐only sample indicate a homogeneous distribution of the Cs, Pb, Cd, and Br elements (Figure S9a, Supporting Information). On the other hand, on the mapping images collected on specific areas of the CsPb_0.44_Cd_0.56_Br_3_ NCs sample, that is, nanocubes and NRs as is noted by green and red squares in Figure S9b, Supporting Information, one can recognize a higher content for the Cd^2+^ cations in the NRs as compared with the nanocubes, and vice versa for Pb^2+^ cations. It appears that the high Cd content in the alloyed CsPb_1‐_ *_x_*Cd*_x_*Br_3_ perovskite NCs is the crucial factor to promote the formation of their rod‐shaped morphology.

We have further addressed the optical properties of the alloyed CsPb_1‐_ *_x_*Cd*_x_*Br_3_ NCs. Their absorption and PL spectra show an obvious blue shift with increasing Cd content (**Figure** **3**a), which is caused by the gradually broadening band gap of the alloy. Bare CsPbBr_3_ NCs have a narrow PL band (full width at half maximum (FWHM) of 16 nm) centered at around 518 nm, which is well in agreement with literature data on these perovskite NCs,^[^ ^2^, ^14^ ^]^ while the PL band of CsPb_0.08_Cd_0.92_Br_3_ and CsPb_0.04_Cd_0.96_Br_3_ NRs blue‐shifted to 460 and 455 nm, respectively, and became broader (FWHM = 32 and 33 nm, respectively). Such a PL broadening is also observed for the CsPb_1‐_ *_x_*Cd*_x_*Br_3_ NCs with the Cd content in the range from *x* = 0.20 to *x* = 0.56 (Figure 3a), where the hexagonal structure emerges in the alloyed NCs. The PL band of CsPb_0.90_Cd_0.10_Br_3_ NCs with lower Cd content and the cubic crystal structure still presents a similar FWHM (18 nm) as for the bare CsPbBr_3_ NCs (Figure 3a).

**Figure 3:**
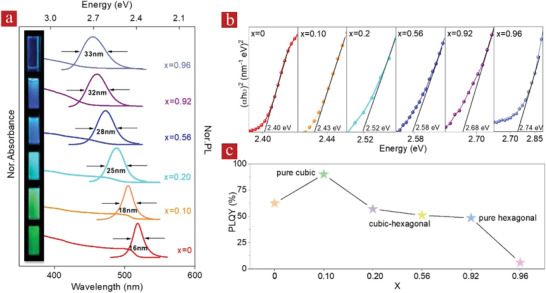
a) Absorption and emission (excitation wavelength 365 nm) spectra of CsPbBr_3_ NCs (*x* = 0) and of alloyed CsPb_1‐_ *_x_*Cd*_x_*Br_3_ NCs with different Cd content (*x*); photographs demonstrating different emission colors of the respective solutions are shown on the left. b) Tauc plots and c) PLQYs of CsPbBr_3_ and alloyed CsPb_1‐_ *_x_*Cd*_x_*Br_3_ NCs with different Cd content (*x*).

Using Tauc plots of (*ahv*)^2^ versus *hv* as presented in Figure 3b, the band gaps of alloyed CsPb_1‐_ *_x_*Cd*_x_*Br_3_ perovskite NCs as a function of Cd content (*x*) have been estimated, which range from 2.40 eV for *x* = 0.10 to 2.74 eV for *x* = 0.96. Trends of the changing band gaps of the alloyed bulk CsPb_1‐x_Cd_x_Br_3_ perovskites as a function of *x* were also simulated by the special quasirandom structures (SQS) method,^[^ ^17^ ^]^ which is used for disordered solid solution structures (the details are presented in the Supporting Information). From the data of SQS simulations (Figure S11, Supporting Information), the CsPb_1‐_ *_x_*Cd*_x_*Br_3_ NCs exhibit a direct band gap, which remains almost constant at lower Cd content (*x* < 0.6). When the Cd content increases from 0.6 to 0.9, the band gap significantly increases, corresponding to the appearance of the 1D hexagonal structure in the alloyed perovskite NCs. SQS simulations performed for the bulk CsPbBr_3_ perovskites with either a 3D cubic structure or bulk CsCdBr_3_ 1D perovskites with an hexagonal crystal structure (Figure S12, Supporting Information) further confirm the above judgment, as they show that the band gap of the 1D hexagonal phase is larger than that of the 3D phase.

Figure 3c summarizes the PLQYs of the alloyed CsPb_1‐_ *_x_*Cd*_x_*Br_3_ perovskite NCs with different values of *x*. The PLQY of the CsPbBr_3_ NCs was 62%; this value increased to impressive 92% for the CsPb_0.90_Cd_0.10_Br_3_ NCs, which demonstrates that the introduction of a small amount of Cd can decrease the number and density of defects, leading to an increased PLQY. Followed by a gradually decreasing PLQY upon further increasing the Cd content, the decrease of PLQY is mainly due to the appearance of the hexagonal crystal phase in the alloyed NCs with the higher Cd content. For CsPb_0.08_Cd_0.92_Br_3_ NRs emitting at 460 nm, the PLQY of 48% was achieved, which is well‐comparable with the PLQY of other blue‐emitting perovskite NRs.^[^ ^8b^ ^]^ Note that this is a true blue emission at 460 nm that meets the National Television System Committee (NTSC) standard,^[^ ^18^ ^]^ and thus the reported CsPb_0.08_Cd_0.92_Br_3_ NRs are important for future developments of the display technology.^[^ ^18^, ^19^ ^]^

From the PLQYs and the average PL lifetimes (Figure S13a, Supporting Information), radiative and nonradiative recombination rates have been calculated for the alloyed CsPb_1‐_ *_x_*Cd*_x_*Br_3_ NCs with different Cd content (see Supporting Information for details), which are summarized in Table S2, Supporting Information. Samples listed in Table S2, Supporting Information, are CsPbBr_3_ and CsPb_0.90_Cd_0.10_Br_3_ NCs with pure cubic phase, and CsPb_0.08_Cd_0.92_Br_3_ and CsPb_0.04_Cd_0.96_Br_3_ NRs with pure hexagonal phase. The other samples, where there were obvious and different nanoparticle impurities (as evident on the TEMs of those samples), were not selected for PL lifetime analysis. Nonradiative decay rates exhibited little variation for the CsPbBr_3_ NCs, CsPb_0.08_Cd_0.92_Br_3_, and CsPb_0.04_Cd_0.96_Br_3_ NRs, but decreased for the CsPb_0.90_Cd_0.10_Br_3_ NCs, which probably resulted from the modification of defect states upon the introduction of small amounts of Cd^2+^ cations. At the same time, radiative decay rates exhibited stronger change upon varying the Cd content in the alloyed NCs (Table S2, Supporting Information). It increased by 1.8 times for the CsPb_0.90_Cd_0.10_Br_3_ NCs as compared to that in the CsPbBr_3_ NCs, owing to the stronger quantum confinement and exciton binding energy in smaller CsPb_0.90_Cd_0.10_Br_3_ particles (Figure S4, Supporting Information). Upon further increasing the Cd content, the radiative decay rates for the CsPb_0.08_Cd_0.92_Br_3_ and CsPb_0.04_Cd_0.96_Br_3_ alloyed NRs decreased significantly compared with that of the CsPbBr_3_ NCs, which determined the decrease of the PLQYs as shown in Figure 3c. We further calculated the transition matrix elements of the bulk CsPb_1‐_ *_x_*Cd*_x_*Br_3_ perovskites with cubic and hexagonal crystal phase (Figure S13b, Supporting Information), and observed a one order of magnitude decrease of the transition strength for the sample with the hexagonal phase as compared to the sample with the cubic phase. Therefore, the decrease of the radiative decay rates mainly results from the appearance of the hexagonal crystal phase in the alloyed NCs with the higher Cd content.

Capacitor‐like devices were fabricated by sandwiching perovskite NC films between layers of indium tin oxide and gold (inset of **Figure** **4**a). From the current–voltage curves shown in Figure 4a, the resistance (*R*) values were obtained for these devices, which are listed in Table S3, Supporting Information. The electrical conductivity (*σ*) of the perovskite NC films has been calculated using equation $\sigma =\frac{d}{AR}$, where *A* is the device area (4 mm^2^), and the values of *R* and the measured thickness of the perovskite films *d* are provided in Table S3, Supporting Information. For the films made of CsPbBr_3_ and CsCdBr_3_ NCs, the conductivities were 1.04 × 10^−2^ and 1.60 × 10^−1^ S cm^−1^ (Figure 4b; Table S3, Supporting Information), respectively, demonstrating their semiconductor‐like behavior. Films deposited from the alloyed CsPb_1‐_ *_x_*Cd*_x_*Br_3_ NCs were much more conductive, especially those with the higher content of Cd^2+^ cations (≥0.92) (Figure 4a). Conductivities of the films made of CsPb_0.08_Cd_0.92_Br_3_ and CsPb_0.04_Cd_0.96_Br_3_ NRs were 2.20 × 10^−1^ and 2.50 × 10^−1^ S cm^−1^, respectively, which is 20 times higher than that of CsPbBr_3_ NCs. To investigate how the Cd alloying changes the electronic properties of CsPbBr_3_ perovskite NCs, the ultraviolet photoelectron spectra of the alloyed NCs as well as CsPbBr_3_ counterparts were measured and are provided in Figure S14, Supporting Information. Using optical band gaps from the Tauc plots shown in Figure 3b, we determined the energy difference between the Fermi level and the conduction band minimum, which was 0.48 eV for the bare CsPbBr_3_ NCs, and ranged from 0.42 to –0.03 eV for CsPb_1‐_ *_x_*Cd*_x_*Br_3‐_alloyed NCs with different Cd content (*x*) (Figure 4c). This result indicates that the alloyed CsPb_1‐_ *_x_*Cd*_x_*Br_3_ NCs, in particular the CsPb_0.08_Cd_0.92_Br_3_ and CsPb_0.04_Cd_0.96_Br_3_ NRs, exhibit stronger n‐type behavior owing to the considerable increase of the electron concentration caused by Cd alloying in the mixed Pb/Cd alloyed perovskites.^[^ ^20^ ^]^

**Figure 4:**
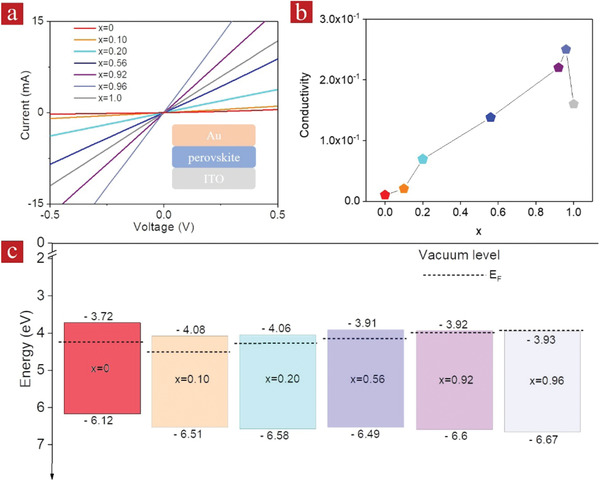
a) Current–voltage curves, b) conductivity, and c) energy band diagrams (with the Fermi levels *E*_F_ indicated by dashed lines) of the films made of CsPbBr_3_ NCs (*x* = 0), CsCdBr_3_ (*x* = 1), and alloyed CsPb_1‐_ *_x_*Cd*_x_*Br_3_ NCs with different Cd content (*x*).

To conclude, we have shown that upon incorporation of sufficient levels of Cd^2+^ cations, the isotropic 3D cubic lattice of CsPbBr_3_ NCs gives way to a transformation to the 1D anisotropic hexagonal lattice, which allows us to realize 1D growth of alloyed CsPb_1‐_ *_x_*Cd*_x_*Br_3_ NRs. We have used DFT calculations to predict the influence of the Cd content on the resulting crystal phase of the alloyed CsPb_1‐_ *_x_*Cd*_x_*Br_3_ NCs. Calculation results and experimental findings of this study indicate that a large excess of Cd^2+^ cations in the reaction environment is the requirement to achieve the 1D hexagonal crystallographic structure of the alloyed CsPb_1‐_ *_x_*Cd*_x_*Br_3_ perovskite NCs. We explored the optical properties of CsPb_1‐_ *_x_*Cd*_x_*Br_3_‐alloyed NCs, whose band gaps can be tailored all across the blue spectral range from 2.40 eV for *x* = 0.1 to 2.74 eV for *x* = 0.96. For the CsPb_0.08_Cd_0.92_Br_3_ NRs emitting blue light at 460 nm, a PL QY of 48% was achieved. We have also shown how the Fermi levels change in the films made of CsPb_1‐_ *_x_*Cd*_x_*Br_3_‐alloyed NCs, where samples with the highest Cd content experienced a corresponding increase of the electron concentration.

## Conflict of Interest

The authors declare no conflict of interest.

## Supporting information

Supporting Information
