# Supplementary material for: Cd‐Rich Alloyed CsPb1‐ xCdxBr3 Perovskite Nanorods with Tunable Blue Emission and Fermi Levels Fabricated through Crystal Phase Engineering
Source: Adv Sci (Weinh). 2020 Jun 17;7(15):2000930. doi: 10.1002/advs.202000930 (PMC7404144; doi:10.1002/advs.202000930)
Supplement: Supplementary file 1 — Supporting Information [file ADVS-7-2000930-s001.pdf]

© 2020 Wiley-VCH GmbH

ADVANCED SCIENCE
Open Access

Supporting Information

for *Adv. Sci.*, DOI: 10.1002/advs.202000930

Cd-Rich Alloyed $CsPb_{1-x}Cd_xBr_3$ Perovskite Nanorods with Tunable Blue Emission and Fermi Levels Fabricated through Crystal Phase Engineering

Jie Guo, Yuhao Fu, Min Lu, Xiaoyu Zhang, Stephen V. Kershaw, Jia Zhang, Shulin Luo, Yanxiu Li, William W. Yu, Andrey L. Rogach, Lijun Zhang*, Xue Bai**

Supporting Information

Cd-Rich Alloyed $CsPb_{1-x}Cd_xBr_3$ Perovskite Nanorods with Tunable Blue Emission and Fermi Levels Fabricated through Crystal Phase Engineering

Jie Guo, Yuhao Fu, Min Lu, Xiaoyu Zhang, Stephen V. Kershaw, Jia Zhang, Shulin Luo, Yanxiu Li, William W. Yu, Andrey L. Rogach, Lijun Zhang*, Xue Bai**

J. Guo, M. Lu, J. Zhang, Prof. X. Bai
State Key Laboratory of Integrated Optoelectronics and College of Electronic Science and Engineering, Jilin University, Changchun 130012, China

Dr. Y. Fu.
State Key Laboratory of Superhard Materials and College of Physics, Jilin University, Changchun 130012, China

Prof. W. W. Yu
Department of Chemistry and Physics, Louisiana State University, Shreveport, LA 71115, USA

Dr. X. Zhang, S. Luo, Prof. L. Zhang
State Key Laboratory of Integrated Optoelectronics, Key Laboratory of Automobile Materials of MOE and College of Materials Science and Engineering, Jilin University, Changchun 130012, China

Y. Li, Dr. S. V. Kershaw, Prof. A. L. Rogach.
Department of Materials Science and Engineering, and Centre for Functional Photonics (CFP), City University of Hong Kong, Kowloon, Hong Kong SAR

*E-mails: andrey.rogach@cityu.edu.hk (ALR); lijun_zhang@jlu.edu.cn (LZ); baix@jlu.edu.cn (XB)

I. First-principles DFT calculations

First-principles density-functional theory (DFT) calculations were performed by using plane-wave pseudopotential methods as implemented in the Vienna *Ab initio* Simulation Package (VASP).[1] The electron-ion interactions were described by the projector augmented wave method[2] with the 5s, 5p, and 6s (Cs), 5d, 6s and 6p (Pb), 4d and 5s (Cd), 4s and 4p (Br) electrons treated explicitly as valence electrons. We have used the generalized gradient approximation formulated by Perdew, Burke, and Ernzerhof (PBE) as the exchange-correlation functional.[3] Kinetic energy cutoff for the plane-wave basis set was at 360 eV. The k-point meshes with a grid spacing of 2π×0.03 Å^{-1} were used for electronic Brillouin zone integration. We explored the phase diagram of $CsPb_{1-x}Cd_xBr_3$ perovskites when alloying on a B-site by the addition of Cd cations by searching all possible structures with 60 atoms. The ground state structures, namely cubic ($Pm\bar{3}m$) phase for $CsPbBr_3$ and hexagonal ($P6_3mmc$) phase for $CsCdBr_3$, were used for evaluating ΔH according to:

$$\Delta \mathrm{H} = \mathrm{H}(CsPb_{1-x}Cd_xBr_3) - (1-\mathrm{x})\mathrm{H}(\mathrm{CsPb}Br_3) - \mathrm{xH}(\mathrm{CsCd}Br_3)$$

The calculated formation enthalpies of the ground state alloy structures at 0K were located near the decomposition line, less than 4 meV per atom. Note that the thermal excitation energy at 300K is about 26 meV, much larger than these energy differences. It implies that the B-site atoms should be disordered at room temperature in these alloy structures. We obtained the disordered structures with the Cd content (x) of 0.1, 0.2, 0.3 and 0.9 and containing 200 atoms in total in each structure by using a special quasi-random structures (SQS) method[4] as implemented in the Alloy Theoretic Automated Toolkit (ATAT) package.[5] We then performed calculations of electronic band structures and absorption spectra of the disordered structures by taking into account the spin-orbit coupling (SOC) effect. To correct the underestimation of bandgaps in common DFT-PBE calculations which arises from self-interaction errors, we remedied calculations of the band structure and absorption spectra by

referencing the bandgaps using the Heyd−Scuseria−Ernzerhof (HSE06) hybrid functional with SOC effect.[6]

II. Experimental Section

Materials: Cs_2CO_3 was obtained from J&K Chemicals. Oleic acid (OA, 90%), 1-octadecene (ODE, 90%) and $CdBr_2$ (ultra dry, 99.999%) were purchased from Alfa Aesar. Oleylamine (OLA, 80-90%) and $PbBr_2$ (99.999%) were purchased from Aladdin. All the chemicals were used as received without any further purification.

Synthesis of $CsPbBr_3$ and $CsCdBr_3$ NCs

Cesium oleate (Cs-OA) was prepared by mixing Cs_2CO_3 (0.814 g), OA (2.5 mL), and ODE (30.0 mL) in a 100 mL three-necked flask, which was degassed and dried under vacuum for 1 h at 120˚C, and then heated to 150˚C under N_2 until a clear solution was obtained.

For the synthesis of $CsPbBr_3$ NCs, 10.0 mL ODE, 0.138 g $PbBr_2$, 1.0 mL OA and 1.0 mL OLA were loaded into a 50 mL three-necked flask, degassed and dried by applying vacuum for 1 h at 120˚C; after the solution became clear, the temperature was raised to 220 ˚C and 1 mL of cesium oleate solution was quickly injected. 5 s later, the reaction mixture was cooled down to room temperature in an ice-water bath. The solution was centrifuged for 10 min at 5000 rpm, and the obtained precipitate was redispersed in 5.0 mL of hexane, centrifuged down for 10 min at 10000 rpm, redispersed in 3.0 mL of hexane, centrifuged down again for 5 min at 12000 rpm, and redispersed in 1.0 mL of hexane.

For the synthesis of $CsCdBr_3$ NCs, the synthetic procedure was the same as the one used to make $CsPbBr_3$ NCs, except for changing 0.138 g $PbBr_2$ for 0.4 g $CdBr_2$.

Synthesis of alloyed $CsPb_{1-x}Cd_xBr_3$ NCs

Taking alloyed $CsPb_{1-x}Cd_xBr_3$ NCs synthesized using a $CdBr_2$/($CdBr_2$ + $PbBr_2$) molar ratio of 50% as an example, 10.0 mL ODE, 0.138 g $PbBr_2$, 0.10 g $CdBr_2$, OA (1.0 mL) and OLA (1.0 mL) were loaded into a 50 mL three-necked flask, degassed and dried by applying vacuum for

1 h at 120˚C; after the solution became clear, the temperature was raised to 220 ˚C and 1 mL of cesium oleate solution was quickly injected. 5 s later, the reaction mixture was cooled down to room temperature in an ice-water bath, and the final product was obtained by applying several centrifugation steps in the same way as described above for the $CsPbBr_3$ NCs. For the other alloyed $CsPb_{1-x}Cd_xBr_3$ NCs, the synthetic procedures were identical, except for changing the $CdBr_2$ amount from 0.1 g to 0.2, 0.3, 0.4 and 0.50 g for the $CdBr_2/(CdBr_2+PbBr_2)$ molar ratios of 67, 75, 80 and 83%, respectively.

Characterization

X-ray diffraction (XRD) patterns were acquired using a Bruker D8 Advance X diffractometer (Cu Kα, λ = 1.5406 Å). Transmission electron microscopy (TEM) and high-resolution TEM (HR-TEM) images with the related elemental mapping and EDX analysis were performed on a FEI Tecnai F20 microscope. X-ray photoelectron spectroscopy (XPS) was conducted on an ESCALAB250 spectrometer. Inductively coupled plasma mass-spectrometry (ICP-MS) was carried out on a Thermo iCAP Qc machine. UV-vis absorption spectra were measured on a Shimadzu UV-2550 spectrophotometer, and photoluminescence (PL) spectra on an Ocean Optics spectrometer. Absolute PL quantum yields (QYs) of the samples were obtained on a fluorescence spectrometer (FLS920P, Edinburgh Instruments) equipped with an integrating sphere. Time-resolved PL lifetime measurements were carried out using a time-correlated single-photon counting (TCSPC) lifetime spectroscopy system with a picosecond pulsed diode laser (EPL-365 nm) as the single wavelength excitation light source. A Keithley 2612B source meter was used to measure the current-voltage characteristics of perovskite films. Ultraviolet photoelectron spectroscopy (UPS) was performed on a PREVAC system. Thickness of the films was determined by a XP-2 step profiler. Conductivity measurements were performed on capacitor-like devices (ITO/perovskite/Au). Cleaned ITO substrates were treated by UV-ozone for 10 min, transferred into a glovebox, and the perovskite layer was

spin cast from solution perovskite NCs in hexane. Au layers were deposited on top by thermal evaporation in a vacuum deposition chamber (1×10^{-7} Torr).

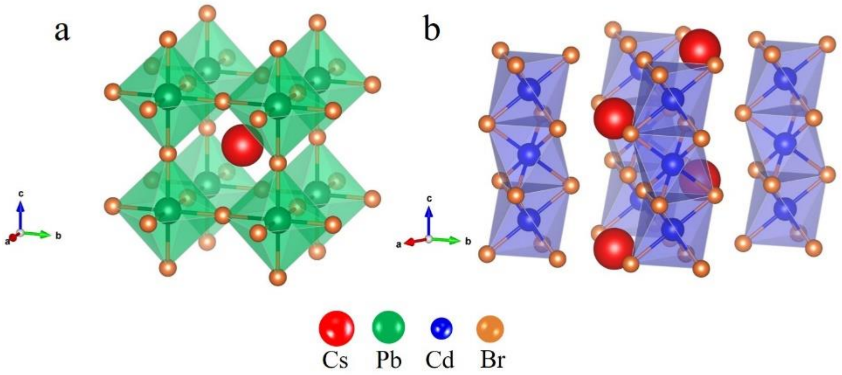

Figure S1. Schematic illustration of the crystal structure of (a) 3D cubic phase of $CsPbBr_3$, and (b) 1D hexagonal phase of $CsCdBr_3$.

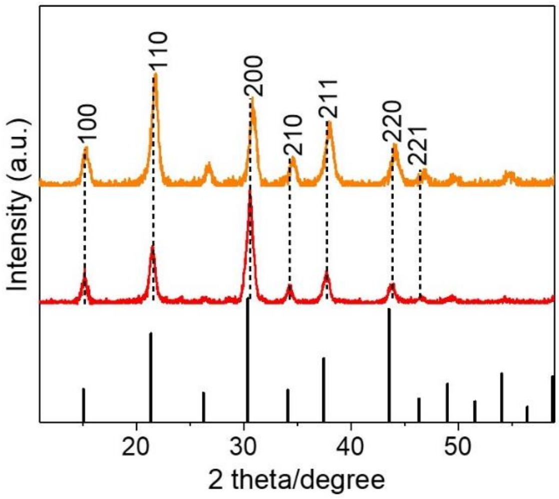

Figure S2. XRD patterns of $CsPbBr_3$ NCs (red) and alloyed $CsPb_{0.90}Cd_{0.10}Br_3$ NCs (orange). The line spectrum at the bottom provides the XRD reflexes of the cubic $CsPbBr_3$ phase (PDF #74-412).

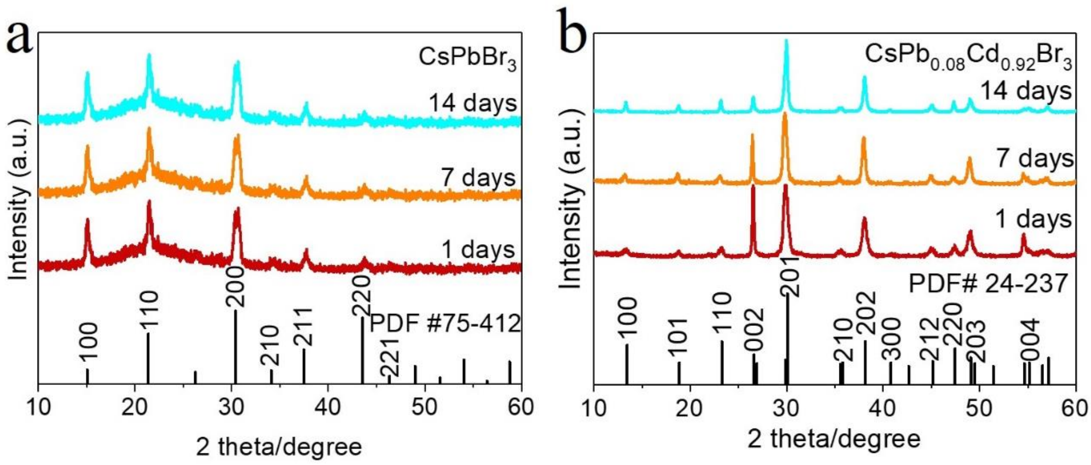

Figure S3. XRD patterns of (a) 3D $CsPbBr_3$ NCs with the cubic crystal structure, and (b) 1D $CsPb_{0.08}Cd_{0.92}Br_3$ NRs with hexagonal crystal structure, which were taken during their storage as films in the air, for up to 14 days.

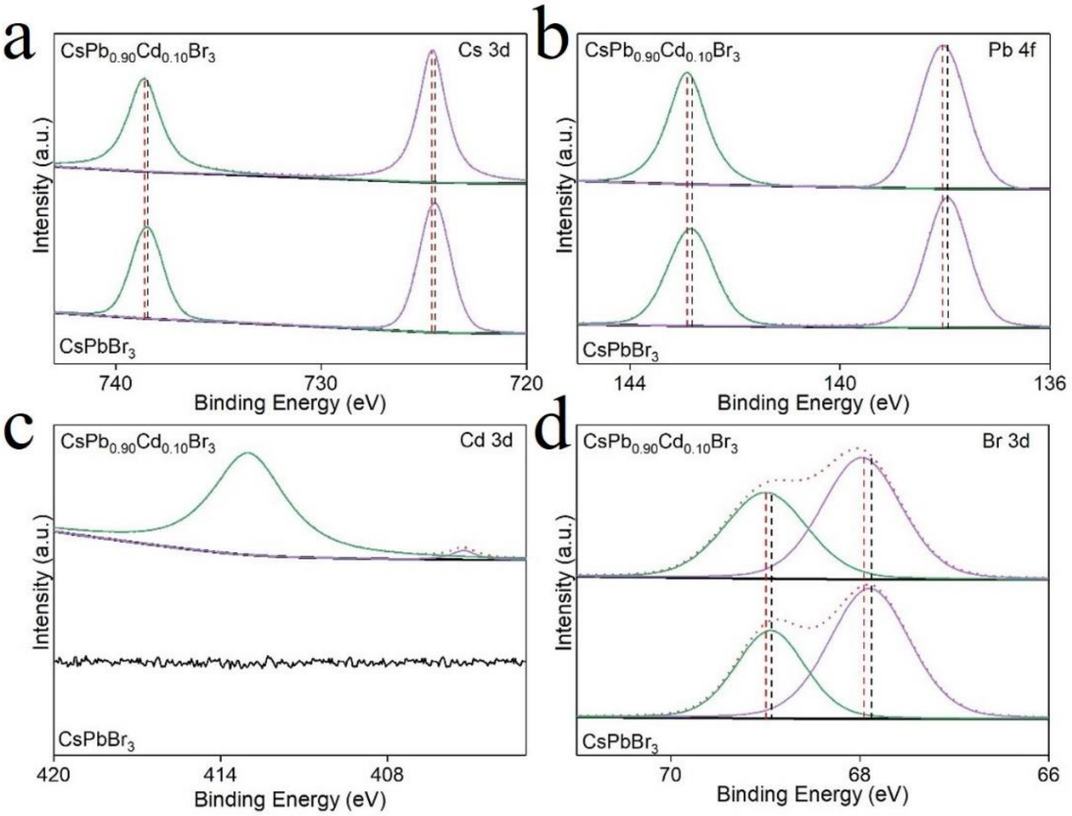

Figure S4. High-resolution XPS spectra for (a) Cs 3d, (b) Pb 4f, (c) Cd 3d and (d) Br 3d in the $CsPbBr_3$ NCs and alloyed $CsPb_{0.90}Cd_{0.10}Br_3$ NCs, both of them with a 3D cubic crystal structure.

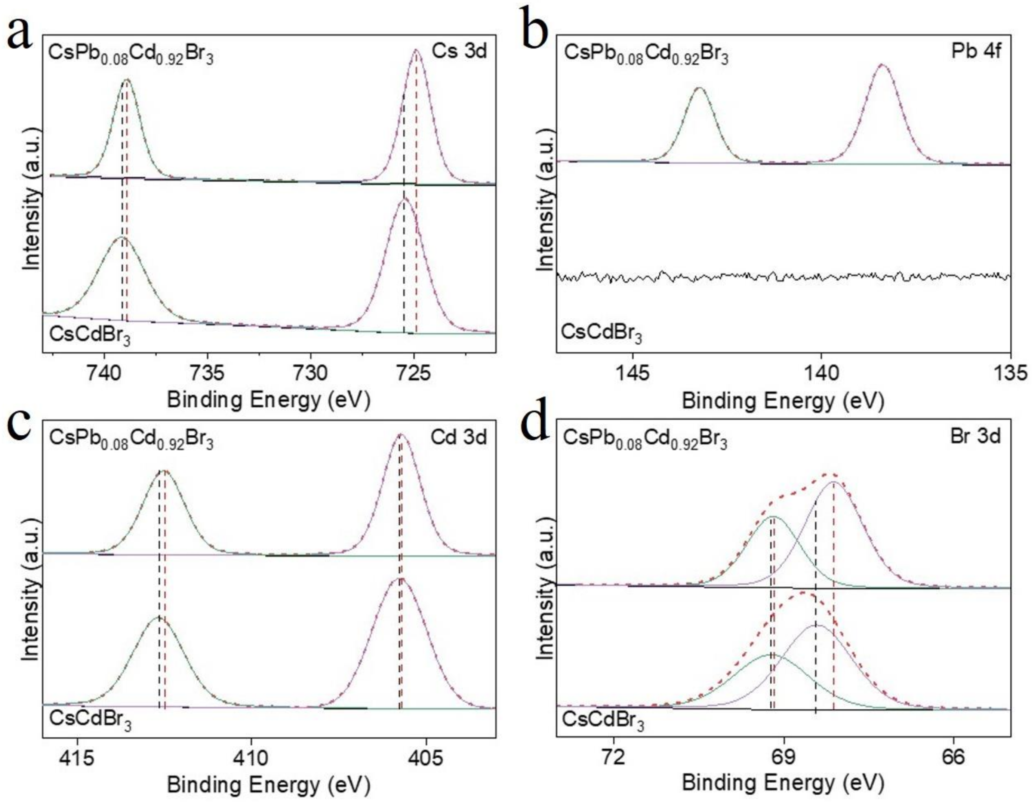

Figure S5. High-resolution XPS spectra for (a) Cs 3d, (b) Pb 4f, (c) Cd 3d and (d) Br 3d in the $CsCdBr_3$ NRs and alloyed $CsPb_{0.08}Cd_{0.92}Br_3$ NRs, both of them with a 1D hexagonal crystal structure.

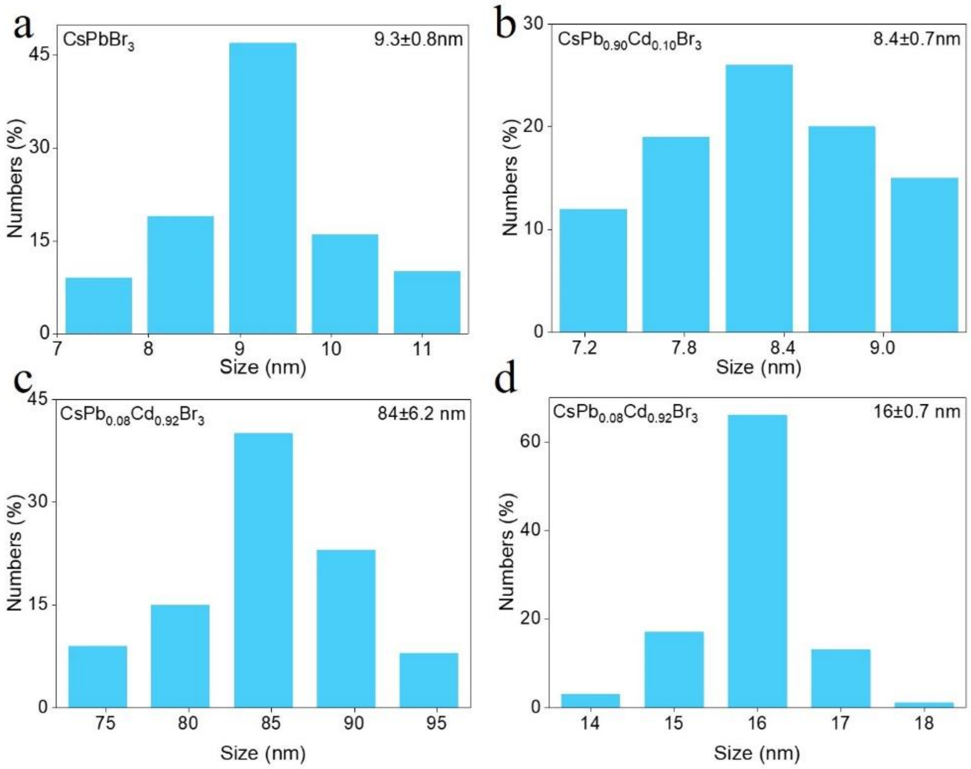

Figure S6. Size histograms of (a) $CsPbBr_3$ and (b) $CsPb_{0.90}Cd_{0.10}Br_3$ NCs. Distributions of (c) lengths and (d) diameters of $CsPb_{0.08}Cd_{0.92}Br_3$ NRs. Average values estimated from these graphs are provided at the upper right corners.

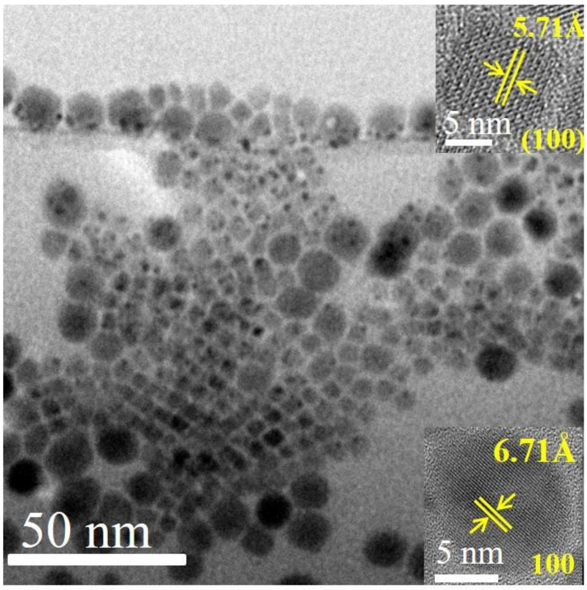

Figure S7. TEM image of $CsPb_{0.80}Cd_{0.20}Br_3$ NCs. Insets on the top and the bottom show HR-TEM images of a nanocube and a spherical nanoparticle, respectively. Scale bars are 50 nm for the main frame and 5 nm for insets.

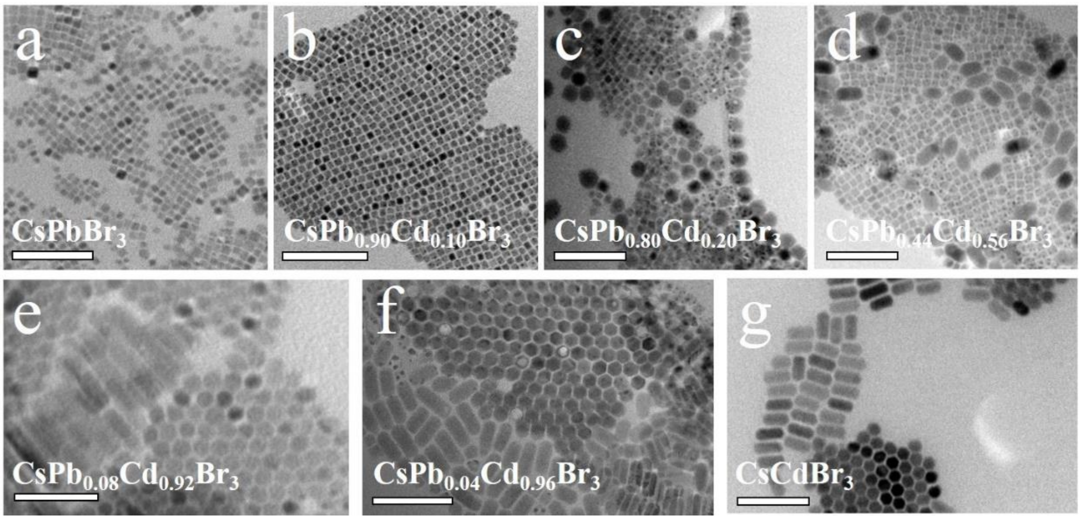

Figure S8. Large area TEM image of the $CsPbBr_3$ NCs and $CsCdBr_3$ NRs, and alloyed $CsPb_{1-x}Cd_xBr_3$ NCs with different Cd contents (x). Scale bars are 100 nm.

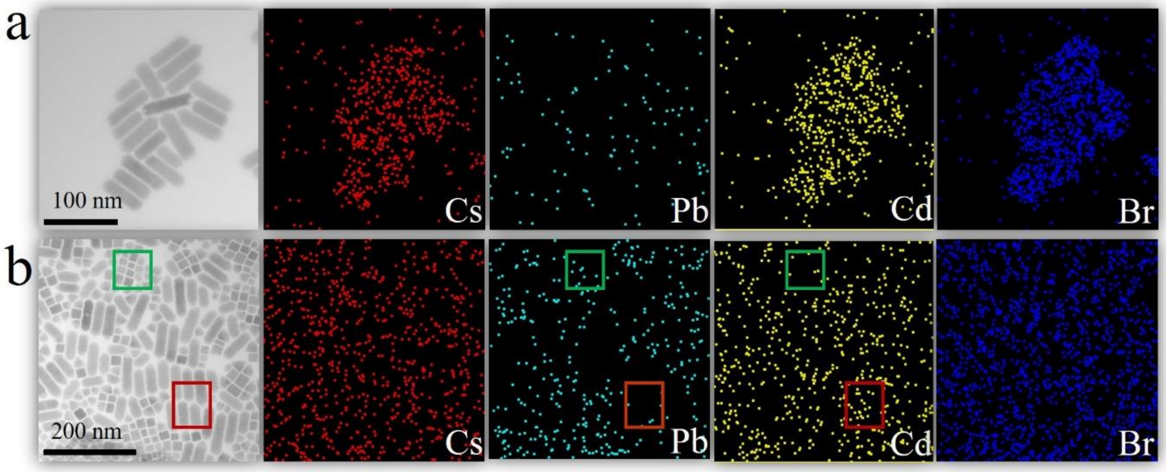

Figure S9. TEM images and respective elemental mapping for Cs, Pb, Cd and Br elements in (a) $CsPb_{0.08}Cd_{0.92}Br_3$ NRs, and (b) $CsPb_{0.44}Cd_{0.56}Br_3$ NCs. In the frame (b), green and red squares indicate the areas which contain nanocubes and NRs, respectively.

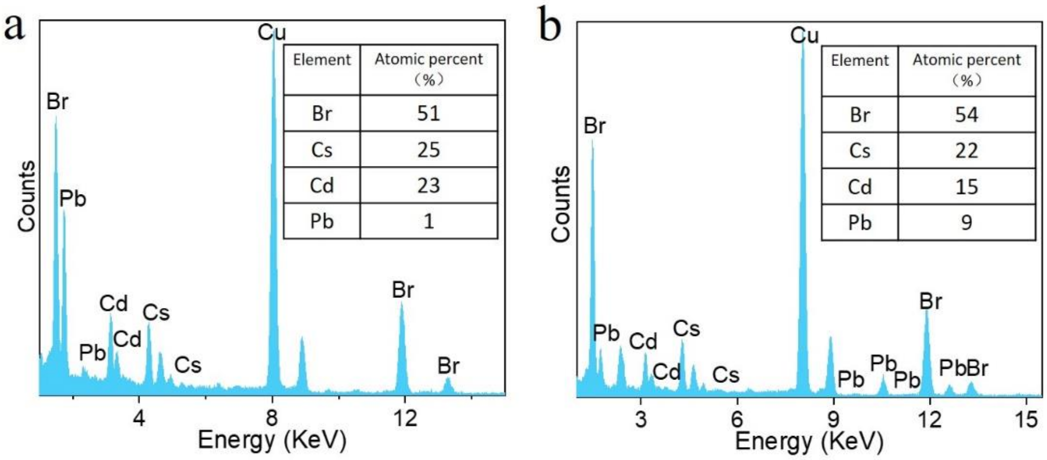

Figure S10. EDX elemental analysis for (a) $CsPb_{0.08}Cd_{0.92}Br_3$ NRs, and (b) $CsPb_{0.44}Cd_{0.56}Br_3$ NCs.

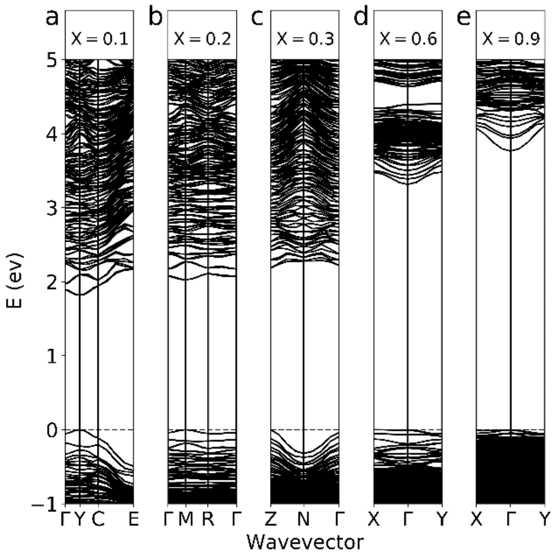

Figure S11. Calculated band structures of the bulk alloyed $CsPb_{1-x}Cd_xBr_3$ perovskite with a different Cd content (x).

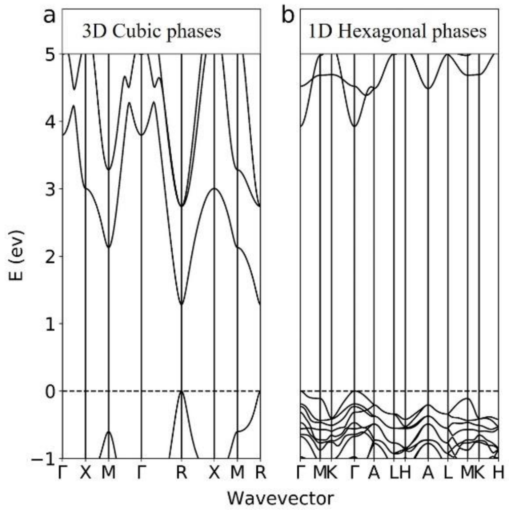

Figure S12. Calculated band structures of (a) bulk $CsPbBr_3$ perovskite with 3D cubic crystal structure, and (b) bulk $CsCdBr_3$ perovskite with 1D hexagonal crystal structure.

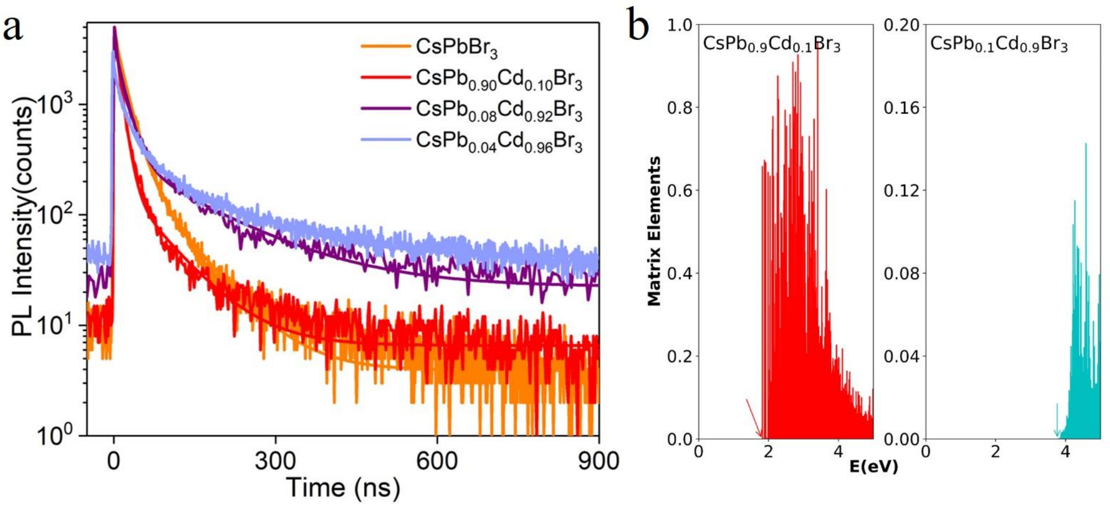

Figure S13. (a) PL decays of $CsPbBr_3$ NCs, $CsPb_{0.90}Cd_{0.10}$ Br_3 NCs, $CsPb_{0.08}Cd_{0.92}Br_3$ NRs and $CsPb_{0.04}Cd_{0.96}Br_3$ NRs. (b) Calculated transition matrix elements of the bulk $CsPb_{0.9}Cd_{0.1}Br_3$ perovskites with 3D cubic crystal structure (left), and of $CsPb_{0.1}Cd_{0.9}Br_3$ perovskites with 1D hexagonal crystal structure (right).

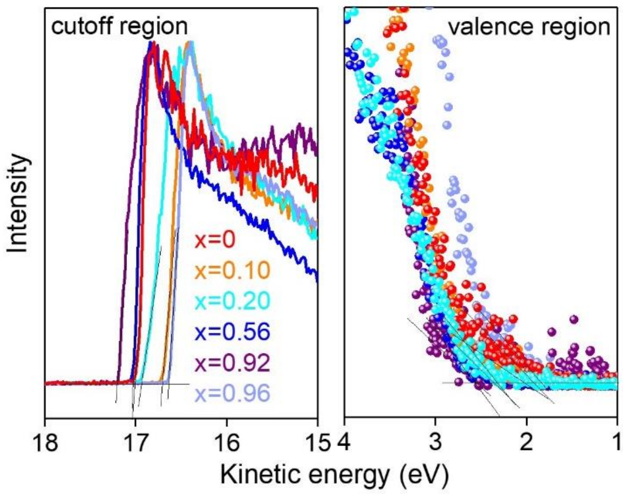

Figure S14. UPS spectra of $CsPbBr_3$ NCs and alloyed $CsPb_{1-x}Cd_xBr_3$ NCs with different Cd content (x) deposited on ITO glass substrates.

Table S1. Calculation results for the lattice constants of the $CsPbBr_3$ NCs, $CsCdBr_3$ NRs, and alloyed $CsPb_{1-x}Cd_xBr_3$ NCs with different Cd content (x).

Cubic phases										
Crystalline planes			(100)	(110)	(111)	(200)	(210)	(211)	(220)	(221)
Lattice distance (Å)	x=0		5.87	4.15	3.39	2.94	2.63	2.40	2.08	1.96
	$CsPb_{1-x}Cd_xBr_3$ NCs	x=0.10	5.75	4.10	3.33	2.89	2.58	2.36	2.05	1.93
		x=0.20	5.71	4.09	N/A	2.88	N/A	2.35	N/A	N/A
			6.71	N/A	3.87	3.38	3.01	N/A	N/A	N/A
		x=0.56	5.70	4.08	N/A	2.87	N/A	N/A	N/A	N/A
			6.70	N/A	3.86	N/A	3.00	2.38	1.87	N/A
		x=0.92	6.67	4.73	3.84	3.37	2.98	2.37	1.86	1.68
		x=0.96	6.65	4.72	3.83	3.36	2.97	2.36	1.85	1.67
	x=1		6.60	4.70	3.82	3.34	2.96	2.35	1.84	1.66
Crystalline planes			(100)	(101)	(110)	(002)	(201)	(202)	(203)	(004)
Hexagonal phases										

Table S2. Data derived from the bi-exponential fitting of PL decay curves (τ_1, f_1, τ_2, f_2, and χ^2), PL average lifetimes (τ_{avg}), radiative decay rates (k_r), PL QYs, and nonradiative decay rates (k_{nr}) of the $CsPbBr_3$ NCs, $CsPb_{0.9}Cd_{0.1}Br$ NCs, $CsPb_{0.08}Cd_{0.92}Br_3$ NRs and $CsPb_{0.04}Cd_{0.96}Br_3$ NRs.

	$CsPbBr_3$	$CsPb_{0.9}Cd_{0.1}Br_3$	$CsPb_{0.08}Cd_{0.92}Br_3$	$CsPb_{0.04}Cd_{0.96}Br_3$
τ_1(ns)	18.4	10.5	17.7	27.4
f_1(%)	71.1	76.0	54.6	46.5
τ_2(ns)	70.4	72.6	145.9	124.5
f_2(%)	28.9	24.0	45.4	53.5
χ^2	0.99	0.99	0.99	0.99
τ_{avg}(ns)	33.0	25.4	76.0	92.0
PL QY (%)	62.5	90.0	48.5	6.0
τ_r (ns)	52.8	28.2	156.7	1533.3
τ_{nr} (ns)	88.0	254.0	147.6	97.9
k_r($\times 10^6$ s^{-1})	19.0	35.5	6.4	0.7
k_{nr}($\times 10^6$ s^{-1})	11.4	3.9	6.8	10.2

PL decay curves shown in Figure S11a can be fitted by biexponential function $I(\mathrm{t}) = I_1 \exp(-t/\tau_1) + I_2 \exp(-t/\tau_2)$, from which average PL lifetimes (τ_{avg}) were calculated, and are listed in the Table S2. Taking into account the PL QYs of the respective samples, radiative/nonradiative lifetimes and decay rates were calculated using the equations below:

Radiative lifetime: $\tau_r = \frac{\tau_{avg}}{QY}$ (1)

Nonradiative lifetime: $\tau_{nr} = \frac{\tau_{avg}}{1-QY}$ (2)

Radiative decay rate: $k_r = \frac{1}{\tau_r}$ (3)

Nonradiative decay rate: $k_{nr} = \frac{1}{\tau_{nr}}$ (4)

Table S3. Resistance, thickness and conductivity of the films deposited from $CsPbBr_3$ NCs, $CsCdBr_3$ NRs, and $CsPb_{1-x}Cd_xBr_3$ NCs synthesized with different Cd content (x). The resistances are determined from the slope of the I-V curves shown in Figure 4a in the main text; thicknesses are measured by a step profiler, and the conductivities are calculated from the formula : $\sigma = \frac{d}{AR}$.

		Resistance (Ω)	Thickness (nm)	Conductivity (S/cm)
x=0		1158.0	480	1.04×10^{-2}
$CsPb_{1-x}Cd_xBr_3$ NCs	x=0.10	480.0	400	2.08×10^{-2}
	x=0.20	129.5	360	6.95×10^{-2}
	x=0.56	58.0	320	1.38×10^{-1}
	x=0.92	29.6	260	2.20×10^{-1}
	x=0.96	20.0	200	2.50×10^{-1}
x=1		42.3	270	1.60×10^{-1}

REFERENCES

[1] a) G. Kresse, J. Furthmüller, *Comput. Mater. Sci.* **1996**, *6*, 15-50; b) G. Kresse, J. Furthmüller, *Phys. Rev. B* **1996**, *54*, 11169-11186.

[2] P. E. Blöchl, *Phys. Rev. B* **1994**, *50*, 17953-17979.

[3] J. P. Perdew, K. Burke, M. Ernzerhof, *Phys. Rev. Lett.* **1996**, *77*, 3865-3868.

[4] A. van de Walle, P. Tiwary, M. de Jong, D. L. Olmsted, M. Asta, A. Dick, D. Shin, Y. Wang, L. Q. Chen, Z. K. Liu, *Calphad* **2013**, *42*, 13-18.

[5] A. van de Walle, M. Asta, G. Ceder, *Calphad* **2002**, *26*, 539-553.

[6] J. Heyd, G. E. Scuseria, *J. Chem. Phys.* **2003**, *118*, 8207-8215.
